# Lipotoxicity and β-Cell Failure in Type 2 Diabetes: Oxidative Stress Linked to NADPH Oxidase and ER Stress

**DOI:** 10.3390/cells10123328

**Published:** 2021-11-26

**Authors:** Eloisa Aparecida Vilas-Boas, Davidson Correa Almeida, Leticia Prates Roma, Fernanda Ortis, Angelo Rafael Carpinelli

**Affiliations:** 1 Department of Physiology and Biophysics, Institute of Biomedical Sciences, University of São Paulo (USP), São Paulo 05508-000, Brazil; 2 Department of Biochemistry, Institute of Chemistry, University of São Paulo (USP), São Paulo 05508-900, Brazil; 3 Department of Cell and Developmental Biology, Institute of Biomedical Sciences, University of São Paulo (USP), São Paulo 05508-000, Brazil; davidson.almeida@usp.br (D.C.A.); fortis@usp.br (F.O.); 4 Center for Human and Molecular Biology (ZHMB), Department of Biophysics, Saarland University, 66424 Homburg, Germany; leticia.prates-roma@uks.eu

**Keywords:** lipotoxicity, pancreatic β-cell, type 2 diabetes, NADPH oxidase, oxidative stress, ER stress

## Abstract

A high caloric intake, rich in saturated fats, greatly contributes to the development of obesity, which is the leading risk factor for type 2 diabetes (T2D). A persistent caloric surplus increases plasma levels of fatty acids (FAs), especially saturated ones, which were shown to negatively impact pancreatic β-cell function and survival in a process called lipotoxicity. Lipotoxicity in β-cells activates different stress pathways, culminating in β-cells dysfunction and death. Among all stresses, endoplasmic reticulum (ER) stress and oxidative stress have been shown to be strongly correlated. One main source of oxidative stress in pancreatic β-cells appears to be the reactive oxygen species producer NADPH oxidase (NOX) enzyme, which has a role in the glucose-stimulated insulin secretion and in the β-cell demise during both T1 and T2D. In this review, we focus on the acute and chronic effects of FAs and the lipotoxicity-induced β-cell failure during T2D development, with special emphasis on the oxidative stress induced by NOX, the ER stress, and the crosstalk between NOX and ER stress.

## 1. Introduction

Type 2 diabetes *mellitus* (T2D) is characterized by a dysfunction in glucose, lipid, and protein metabolism caused by a combination of impaired insulin secretion and insulin sensitivity, resulting in overt hyperglycemia. Of the three major types of diabetes *mellitus* (type 1, type 2, and gestational), T2D is by far the most prevalent, representing more than 90% of cases [1]. Three major abnormalities contribute to hyperglycemia in T2D: decreased insulin sensitivity in (1) muscle; (2) liver; and (3) impaired insulin secretion. Insulin resistance in muscle is characterized by reduced glucose uptake in the postprandial phase, whereas in the liver, the hallmark of insulin resistance is increased hepatic glucose production (HPG) in the face of hyperinsulinemia [2].

Despite T2D having an important genetic component [3,4,5], the recent rise in T2D cases can be mostly attributed to the increase in obesity and lack of physical activity; both being insulin resistance-promoting states [1,2]. Moreover, central (or visceral) adiposity is better linked to insulin resistance and plasma levels of glucose, insulin, cholesterol, triglycerides and high-density lipoprotein cholesterol than total adiposity. In persons with prediabetes (impaired fasting glucose, impaired glucose tolerance, without overt hyperglycemia), insulin resistance is compensated by insulin secretion to maintain normoglycemia. It is only when pancreatic β-cell’s capacity is overwhelmed and insulin secretion fails—via either loss of β-cell function or mass (discussed later)—that overt hyperglycemia and T2D ensue [6,7]. The consequent glucose intolerance can further impair insulin sensitivity and promote micro- and macrovascular complications, typical of T2D.

There is a strong association between lipotoxicity-induced pancreatic β-cell dysfunction and death during the development and progression of T2D [8]. Two main stresses are strongly correlated to this condition, namely the oxidative stress and the endoplasmic reticulum (ER) stress [9]. Of note, both stresses are also involved in the pathogenesis of diabetic complications. In this review, we will discuss the different types of fatty acids (FAs) and their effect on β-cells dysfunction and death, with special interest in the role of one main source of oxidative stress, the NADPH oxidase, and the ER stress.

## 2. Fatty Acids and the Obesity-Related Hyperglycemia in T2D

The alarming increase of obesity and T2D is related to high caloric intake, mainly from foods rich in carbohydrates and saturated fats. This is closely linked to hyperglycemia, where peripheral tissues and pancreatic β-cells play a significant role.

**Figure 1:**
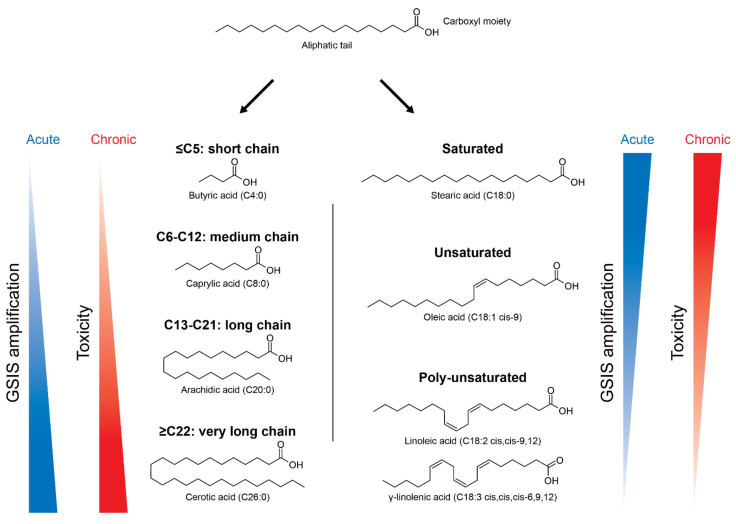
Nomenclature of different types of fatty acids (FAs) and their effects in pancreatic β-cells according to chain length and degree of saturation. FAs are composed by a carboxyl moiety bonded to an aliphatic tail, with variable number of carbons. Depending on the number of carbons, FAs can be divided in short-chain (C ≤ 5), medium-chain (C6-C12), long-chain (C13-C21) and very long-chain (C ≥ 22). They can also be: (i) saturated, with no double bonds, as stearic acid (C18:0); (ii) unsaturated, with one double bond, as oleic acid (C18:1); and (iii) poly-unsaturated, with more than two double bonds, as linoleic acid (C18:2) and γ-linolenic acid (C18:3). FAs are known to increase the glucose-stimulated insulin secretion (GSIS), but can also be toxic in the long-term. The potency of GSIS amplification is positively influenced by the carbon chain length and negatively influenced by the degree of unsaturation.

Lipids are a broader class of organic compounds insoluble to water and with variable structures, including triacylglycerols (TAGs), FAs, cholesterol, etc. [10]. FAs are important structural components of cell membranes and represent a significant metabolic fuel for several cell types. Their molecules are formed by an apolar region, containing a carbon chain with variable number of carbons, bonded to a polar region, which is a carboxyl moiety (Figure 1). According to their chain length, FAs are classified into: short-chain (<C_5_), medium-chain (C_6_-C_12_), long-chain (C_13_-C_21_), and very long-chain (>C_22_) [10] (Figure 1). FAs may also be saturated, with no double bonds in its carbon chain, or unsaturated, containing one or more double bonds. Regarding its nomenclature, there is an abbreviated notation in which the number of carbons and the number of double bonds are written as C18:2, meaning a FA with 18 carbons and 2 double bonds [10]. In addition, the location of the first double bond may also be indicated as 18:2n-6, known as linoleic acid. Different FAs can have beneficial or detrimental effects on pancreatic β-cells’ function, depending on the time of exposure and the type of FA, as discussed later.

**Figure 2:**
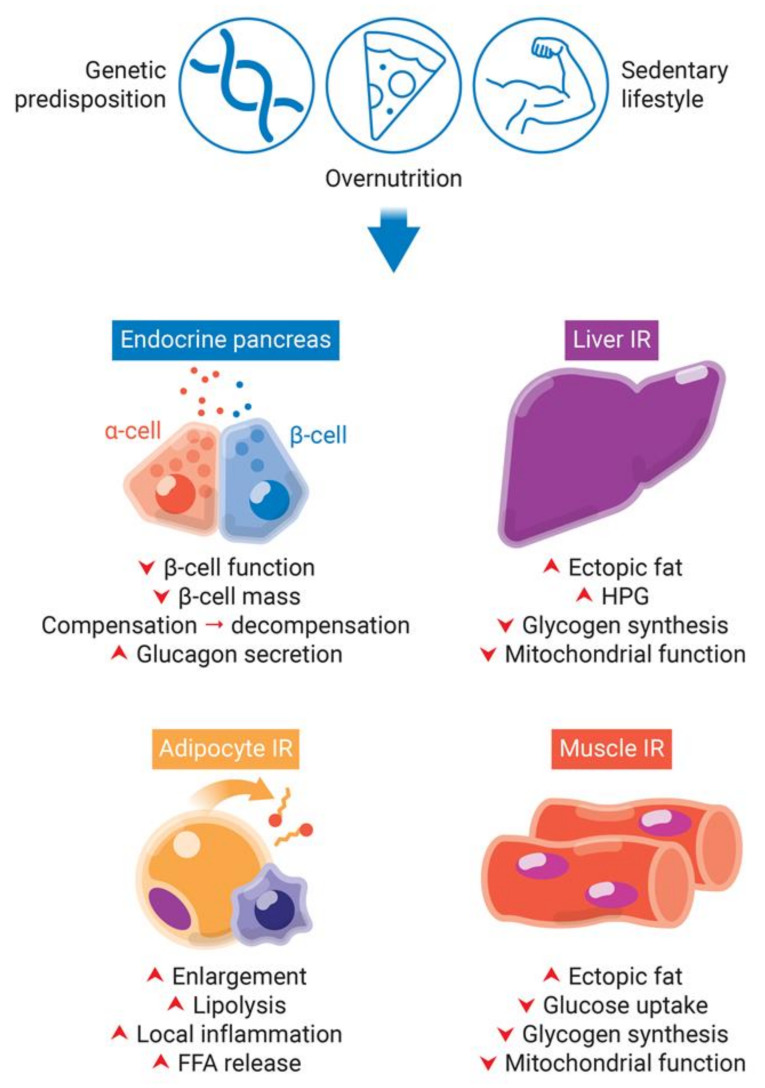
Obesity-induced pancreatic β-cells dysfunction and insulin resistance (IR) in peripheral tissues. Genetic predisposition, overnutrition, and a sedentary lifestyle are involved in obesity and type 2 diabetes development. The lipotoxicity involved in chronic obesity and hyperglycemia leads to an increase of insulin demand due to IR in peripheral tissues. While in a first moment, β-cells try to compensate for IR by increasing insulin synthesis, they start to decompensate in late stages, with decrease of β-cell function and mass. IR in adipocytes eliminate insulin-induced repression of lipolysis. Impaired adipogenesis and dysfunctional hypertrophy of adipocytes increase plasma levels of free fatty acids (FFAs). Additionally, dysfunctional adipose tissue releases increased levels of insulin resistance-inducing pro-inflammatory adipokines, leading to local inflammation. The increased FFAs flux to the liver and skeletal muscle leads to increase of fat deposition, decrease of glycogen synthesis and mitochondrial dysfunction. Additionally, the hallmark of insulin resistance in the liver is increased hepatic glucose production (HPG).

Both saturated and unsaturated FAs are commonly found in the human nutrition and once absorbed in the intestinal tract, they circulate in the blood bonded to plasma proteins. FAs are imported into cells by free diffusion and/or through membrane FA transporters, such as CD36 [11,12,13]. High intake of FAs, especially saturated ones, leads to decreased insulin sensitivity in peripheral tissues (Figure 2).

The major abnormality of muscle insulin resistance is decreased glucose uptake and glycogen synthesis [14] (Figure 2). This is caused by increased delivery of free fatty acids (FFAs) to muscles, due to obesity and consequently elevated plasma levels of TAGs, FFAs, and very low-density lipoproteins (VLDLs), together with increased intramuscular and intramyocellular TAGs levels (ectopic fat deposition) (Figure 2). In fact, intramuscular and intramyocellular TAGs are a better predictor of insulin resistance than total adiposity and circulating plasma FFAs. Additionally, muscle lipid accumulation and insulin resistance are associated with decreased lipid oxidation mediated by reactive oxygen species (ROS)-induced mitochondrial damage and age-related reduction in mitochondrial biogenesis [15,16].

Under normal conditions, the liver is responsible for insulin-induced reduction of glucose production and insulin-stimulated increase of glycogen synthesis after caloric intake. The increased FFAs flux to the liver and the consequent steatosis (increased hepatic ectopic lipid deposition) induces hepatic insulin resistance in a similar fashion to muscle [14] (Figure 2). Lipid-induced hepatic insulin resistance is marked by decreased insulin-stimulated glycogen synthase flux and impaired insulin-induced inhibition of glucose production [14] (Figure 2).

Adipocyte dysfunction can contribute to insulin resistance and impaired insulin secretion. Insulin resistance in adipocytes eliminate insulin-induced repression of lipolysis (Figure 2). This is associated with increased plasma levels of FFAs [17]. Impaired adipogenesis and dysfunctional hypertrophy of adipocytes in situations of persistent caloric surplus also increase plasma FFAs [18] (Figure 2). Furthermore, enlarged adipocytes are more insulin resistant and have limited storage capacity. The consequent increase in the release of circulating FFAs will in turn contribute to ectopic fat deposition and hepatic and muscle insulin resistance, as discussed above [2]. Additionally, dysfunctional adipose tissue releases increased levels of insulin resistance-inducing pro-inflammatory adipokines and decreased levels of insulin-sensitizing anti-inflammatory adipokines, a phenomenon termed “subclinical inflammation”. The release of pro-inflammatory adipokines (e.g., TNF, IL-6, MCP-1) from adipocytes, preadipocytes and resident macrophages is increased during obesity; while the release of anti-inflammatory factors (e.g., adiponectin) is decreased in the same state.

## 3. Acute Versus Chronic Effects of FAs in Pancreatic β-Cells

### 3.1. FAs-Increased Glucose-Stimulated Insulin Secretion (GSIS)

The effects of several FAs on pancreatic β-cell function are pleiotropic and depend on the type of FA, with respect to their degree of unsaturation and chain length, in addition to the concentration and time of exposure [19,20,21,22] (Figure 1). Once inside the β-cell, FAs are esterified to form long-chain acyl-CoAs (LC-CoA) by acyl-CoA synthetase. In low glucose conditions, FAs represent an important source of ATP, and thus they are transported to mitochondria or peroxisomes to be oxidized in acetyl-CoA, in a process known as β-oxidation [23]. Very long-chain FAs are β-oxidized in peroxisomes, whereas short-chain, medium-chain, and long-chain FAs are preferentially β-oxidized in mitochondria [23,24]. After the β-oxidation, acetyl-CoA is fully oxidized to CO_2_ in the Krebs Cycle and the electrons generated are transferred to O_2_ via the electron transport chain, supplying the energy to generate ATP by oxidative phosphorylation.

In high glucose conditions, FAs no longer represent the major fuel and their oxidation is downregulated. The transport of LC-CoA to the mitochondria is inhibited and the subsequent increase of cytosolic LC-CoA leads to the activation of PKC, which leads to the phosphorylation of proteins involved in the exocytosis of insulin granules [25,26,27,28,29]. Thus, acute exposure to long-chain FAs is known to increase GSIS of pancreatic β-cells. Besides, it has been previously shown that under certain circumstances, such as in fasted states, FAs are critically required for an efficient GSIS [30]. The potency of GSIS amplification is positively influenced by the carbon chain length and negatively influenced by the degree of unsaturation [27,31] (Figure 1).

Another mechanism by which FAs amplify GSIS is via activation of G-protein coupled receptors, known as GPRs [32,33,34,35]. There are several types of GPRs activated by FAs with different carbon chain lengths. One of the most studied is GPR40, activated by medium- and long-chain FAs, saturated or unsaturated, such as palmitic (C16:0) and oleic (C18:1n9) acids [26,32,34]. The binding of the FA to GPR40 activates the Gαq protein, causing phospholipase C (PLC) stimulation, leading to the formation of DAG and inositol-1,3-biphosphate (IP3), which activates PKC and mobilizes calcium from the ER, respectively [34,36,37,38,39]. GPR40 is expressed in rat and mice pancreatic islets [32] and in insulin-secreting cell lines [25,26]. It has been shown that human pancreatic islets of T2D patients had decreased expression of GPR40 [40] and obese individuals had higher mutation frequency of GPR40, leading to impaired intracellular calcium influx and, consequently, impaired insulin secretion [41]. Thus, because of its role in GSIS amplification, GPR40 has been studied as a promising target in the development of drugs for the treatment of T2D [32,42,43,44,45,46,47,48].

### 3.2. FAs-Induced Lipotoxicity in β-Cells

Despite the beneficial acute effects in GSIS potentiation, various studies have shown that T2D patients present high levels of plasma FFAs [49,50,51] and that deleterious parameters related to this condition, such as impaired insulin secretion and glucose intolerance are associated with the elevated levels of saturated FAs, including palmitic acid (C16:0) and stearic acid (C18:0) [52,53,54].

In fact, high concentrations of glucose and lipids have negative impacts on β-cell function and mass, phenomena referred to as glucotoxicity and lipotoxicity, respectively. Since hyperglycemia and hyperlipidemia commonly coexist in T2D and synergistically induce β-cell dysfunction and death, the term glucolipotoxicity is often preferred. Decreased insulin expression, reduced GSIS and β-cell apoptosis are the major manifestations of glucolipotoxicity and they involve many pathways mediated by a myriad of factors, excellently reviewed elsewhere [8,55,56]. Importantly, and as previously discussed, quality, and not only quantity, of FFAs determines glucolipotoxicity: Different degrees of saturation have different effects on dysfunction, with saturated FAs, as palmitic acid, having the worst effects and the highest hazard ratio for T2D [8]. Additionally, glucose is permissive to glucolipotoxicity by means of modulation of FAs metabolism: High glucose diverts FAs way from β-oxidation and towards esterification. However, it is unlikely that, being inert, triglyceride accumulation itself is the molecular mechanism of glucolipotoxicity, being rather a marker of this phenomenon, and many intermediates of esterification have been implicated in β-cell dysfunction [55,56].

It is important to recognize that glucolipotoxicity might represent one of the stages of β-cell response to insulin resistance and hyperglycemia in a spectrum ranging from glucolipoadaptation, to glucolipotoxicity, to β-cell failure [55]. Under this framework, insulin resistance would be compensated by increased β-cell function—termed “glucolipoadaptation” by Prentki and Nolan [57]—to maintain normoglycemia [55]. This response consists in increased β-cell mass, insulin biosynthesis and secretion, likely relying on enhanced responsiveness to FFAs. In genetically predisposed individuals, however, β-cells have limited capacity to compensate for increasing insulin demand resulting from insulin resistance, hyperglycemia and hyperlipidemia. Hence, glucolipotoxicity plays a major role at this stage. β-cells begin to decompensate insulin resistance: Insulin expression and secretion and β-cell mass all start to decline, via the mechanisms described above and, consequently, glycemia and lipidemia start to rise. Finally, chronic hyperglycemia and hyperlipemia will further promote β-cell dysfunction and death and contribute to overt T2D [55,56].

Several in vitro and in vivo studies show that chronic exposure to high levels of saturated FAs appear to be highly detrimental to β-cells. They may cause β-cell dysfunction with reduced insulin biosynthesis [58,59,60], reduced insulin secretion [61,62], and induction of apoptosis [63,64]. As previously discussed, these deleterious effects due to chronic exposure to FAs are known as lipotoxicity [8,65]. In contrast, several previous reports show that long-chain monounsaturated FAs (MUFAs), such as oleic acid (C18:1n9), and long-chain polyunsaturated FAs (PUFAs), such as omega 3 eicosapentaenoic acid (EPA) and docosahexaenoic acid (DHA), not only present a mild or absent toxicity for the β-cell function [64], but also mitigate the toxic effects of saturated FAs [66,67,68,69].

To date, several in vitro studies have focused on MUFAs and PUFAs and their powerful cytoprotective effects in β-cells. For instance, the in vitro incubation of a human pancreatic β-cell line with the saturated palmitic and stearic acids, and not with the unsaturated palmitoleic and oleic acids, induced cell death [70]. Moreover, long-chain MUFAs inhibited the palmitate-induced toxicity in a rat β-cell line in a dose-dependent manner with a defined potency order: C18:1 (oleic acid) > C16:1 (palmitoleic acid) > C14:1 (myristoleic acid) [71]. Of note, the configuration of the double bond was also important with cis geometric configuration, which means that the hydrogen atoms are located at the same size of the double bond, being more potent than trans forms [71]. In addition to preserve β-cell viability, MUFAs and PUFAs also counteracted the saturated FAs-induced impairment in GSIS in mice and human islets in vitro [67,72]. Interestingly, it has also been shown that unsaturated FAs, such as oleic and palmitoleic acids, were able to protect against apoptosis of a β-cell line induced not only by saturated FAs, but also by other apoptotic inducers, such as serum withdrawal and proinflammatory cytokines [73].

During T2D, although loss of β-cell mass may occur over the years, the impairment in insulin secretion is essentially due to β-cell dysfunction [74,75]. Inflammation, oxidative stress, mitochondrial dysfunction, ER stress, and impaired autophagy are examples of mechanisms activated during glucolipotoxicity in β-cells. Glucose and FFAs can inhibit insulin promotor activity, an effect mediated by activation of extracellular signal-regulated kinases 1/2 (ERK1/2), JNK, and Per-Arnt-Sim kinase (PASK), resulting in inhibition of transcription factors crucial for β-cell development and differentiation, such as pancreas/duodenal homeobox factor-1 (PDX-1) and especially v-maf musculoaponeurotic fibrosarcoma oncogene homologue A (MafA). Structural disorganization of insulin granules and Ca^2+^ channels and PKCε activity are implicated in FFAs inhibition of GSIS.

During T2D development, the chronic exposure to glucose and saturated FAs, and the high demand for insulin, leads to an increase of misfolded and unfolded proteins in the ER lumen, due to the high demand for protein synthesis and due to Ca^2+^ disbalance in the ER, inducing ER stress. In addition, there is an increase of ROS production in β-cells and a consequent oxidative stress [9]. Both stresses are interconnected and can initiate the production of chemokines and inflammatory cytokines, which attract and activate immune cells in the islet microenvironment [9]. The exacerbation of this condition leads to disturbance of secretory function, culminating in β-cell apoptosis [9,76].

The lipotoxicity-induced activation of oxidative and ER stresses is also dependent on the type of FA. In this regard, a recent study with EndoC-βH1 human β-cells exposed to different types of saturated or unsaturated long-chain (LC) and very long-chain (VLC) FAs showed a very evident relationship between the degree of saturation and chain length with lipotoxicity [77]. As expected, lipotoxicity intensified with increasing chain length, with VLCs being the most toxic and polyunsaturated being non-toxic [77]. Moreover, VLC saturated or unsaturated FAs greatly increased ROS formation in both peroxisomes and mitochondria, while LC saturated or unsaturated only slightly influenced ROS formation in the peroxisomes, and interestingly, only saturated VLC FFAs activated ER stress [77].

## 4. Oxidative Stress in Lipotoxicity

### 4.1. Reactive Oxygen Species (ROS): Duality of Effects

Oxygen is vital for the generation of energy, as it is the final acceptor of electrons and hydrogen ions during energy metabolism, allowing for an efficient ATP production in mitochondrial oxidative phosphorylation. Thus, redox reactions are fundamental for the maintenance of life, through respiration, metabolism, and energy supply. However, when using oxygen, cells can generate reactive species.

Reactive species can contain oxygen (ROS) or nitrogen (RNS) and are chemically reactive molecules formed by cells in redox reactions during normal or altered aerobic metabolism. ROS and RNS are ubiquitous molecules comprising: (i) free radicals, which have an oxygen or nitrogen atom with unpaired electrons in its outermost shell (valence shell), such as nitric oxide (NO^•^), superoxide (O_2_^•−^), and hydroxyl radical (OH^•^) and (ii) non-radical reactive species, such as hydrogen peroxide (H_2_O_2_) [72]. ROS and RNS can also be divided between anions, such as O_2_^•−^ and peroxynitrite (ONOO^−^), and non-anions, such as H_2_O_2_ [78].

ROS produced by immune cells are of great importance for host defense, to eliminate bacteria and other pathogens [79,80,81]. In addition, ROS are important second messengers, acting as signaling molecules in several cell types [82]. Consequently, ROS have several implications for signal transduction, contributing to cell proliferation, differentiation, migration, and survival [82,83,84]. The participation of ROS in intercellular and intracellular signaling is named redox signaling. In pancreatic β-cells, ROS are important second messengers in GSIS [85,86,87]. In this regard, the redox signaling has recently been established not only as an important, but crucial requirement for proper GSIS, since an increase in ATP along with an increase in H_2_O_2_ are essential in this process [87,88].

H_2_O_2_ is known to be the main ROS involved in redox signaling [89] and its intracellular actions occur mainly through oxidation of thiol groups of target-molecules. Most modifications alter conformation and interactivity of those target-molecules, contributing to their biological effects. However, due to its reactive nature, high concentrations of H_2_O_2_ can lead to additional and irreversible oxidations, leading to permanent alterations and damage to several molecules. Different reactive species may also react with each other and form other species, as in the case of ONOO^−^, generated from the reaction between NO^•^ and O_2_^•−^. ONOO^−^ is extremely reactive, being a potent inducer of cell death [90].

Our organism is, in general, able to counteract the possible damages caused by ROS and RNS through an antioxidant defense system present in cells. Among the known antioxidant enzymes, we have the one responsible for the conversion of O_2_^•−^ in H_2_O_2_, called superoxide dismutase (SOD) [91]. H_2_O_2_ can be eliminated through the action of several enzymes, such as catalase, glutathione peroxidase, thioredoxin, glutaredoxin, and peroxiredoxin.

In situations where there is a high or too prolonged production of ROS/RNS and/or the antioxidant defense systems are not able to counteract this production, we may have a situation known as oxidative or nitrosative stress, leading to disruption of redox signaling, uncontrolled spread of oxidative damage, and cell repair overload. The consequences of oxidative stress include injury, senescence, and cell death. Notably, oxidative stress is one of the causes leading to β-cell dysfunction and death during T2D [92,93].

In comparison to other mammalian cell types, pancreatic β-cells present low expression of classical antioxidant enzymes responsible for the elimination of H_2_O_2_, such as catalase and glutathione peroxidase [94,95,96]. This fact raises the hypothesis that pancreatic β-cells are particularly sensitive to sustained elevation of ROS, making them more vulnerable to oxidative stress [94,95,96,97]. Therefore, under conditions of sustained activation of intracellular reactive species production, β-cells would quickly undergo oxidative stress and failure [96]. Of note, the expression and activity of SOD is not significantly different in β-cells in comparison to other tissues [95], favoring the accumulation of H_2_O_2_, which may be convenient for enabling the use of acute H_2_O_2_ as a second messenger. Thus, this classical view of β-cells’ vulnerability does not seem plausible since these cells are highly specialized and allow for an effective coupling of oxidative phosphorylation with GSIS. Actually, β-cells express thioredoxin, glutaredoxin, and peroxiredoxin isoforms [98], and it was recently shown that peroxiredoxin/thioredoxin are important in the elimination of H_2_O_2_ at the micromolar range in these cells [99]. Moreover, thioredoxin, glutaredoxin, and peroxiredoxin are capable of redox relays, which are defined as the transfer of the redox information forward, allowing for conducting and spreading redox signals.

On the other hand, although these data suggest that β-cells are not as vulnerable to oxidative damage as initially thought, chronic substrate overload, as seen in T2D, and persistent prooxidative environment can disrupt the redox homeostasis. Experimental in vivo and in vitro evidence have pointed out to a close link between oxidative stress and T2D. It has been recently shown that T2D patients present dysregulation, up- or downregulation, of nine genes involved in redox balance [100]. Moreover, the long-term exposure of β-cells to high glucose and saturated FAs, as observed in T2D, leads to impairment in insulin secretion, decrease of insulin gene expression and β-cell death [56]. Chronic in vitro exposure to high glucose and/or palmitate led to impaired insulin secretion of different β-cell lines, derived from rodents or humans (INS-1 832/13 and 1.1E7) [101,102]. Although not all the specific mechanisms involved are completely understood, increased levels of ROS have been linked to β-cell dysfunction and death in conditions that mimic T2D [103,104,105,106,107].

ROS are normally short-lived species [78] and, therefore, must be more relevant specially in sites close to their production. There are several sources of reactive species, such as complexes I and III of the electron transport chain of the mitochondrial matrix, peroxisomes, xanthine oxidase, lipid peroxidases, ER, enzymes of the cytochrome P450, and the NADPH oxidases (NOX) [108,109,110,111,112,113]. ROS are normally produced as byproducts. However, the only source whose sole function is to produce ROS are the NOX enzymes.

### 4.2. NOX in Lipotoxicity

**Figure 3:**
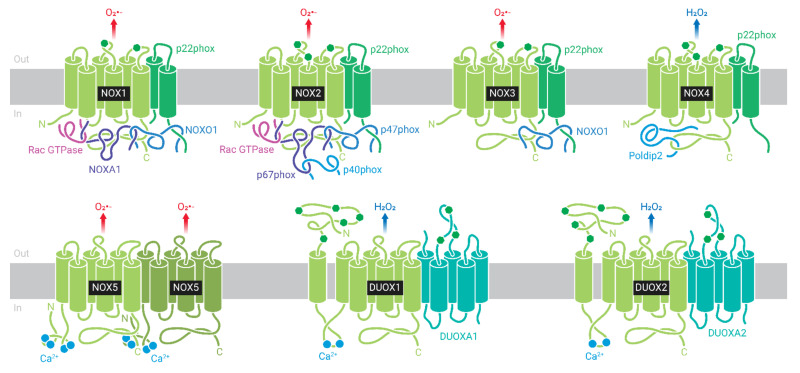
NADPH oxidase (NOX) family: NOX1, NOX2, NOX3, NOX4, NOX5, DUOX1, and DUOX2. NOX1-4 are stabilized by p22^phox^ subunit. NOX1 is activated by NOXO1/NOXA1 and Rac subunits, and NOX2 by p47^phox^/p67^phox^/p40^phox^ and Rac. NOX3 has constitutive activity, but binding to NOXO1/NOXA1 increases reactive oxygen species (ROS) production. NOX4 is constitutively activated. NOX4, NOX5, and DUOX1-2 do not interact with regulatory subunits. NOX5 and DUOX1-2 do not depend on p22^phox^ for stabilization and are activated by Ca^2+^. NOX1, NOX2, NOX3, and NOX5 produce superoxide (O_2_^•−^), and NOX4 and DUOX1-2 produce hydrogen peroxide (H_2_O_2_).

NOX are enzymatic complexes, formed by multiple protein subunits, and are responsible for production of O_2_^•−^ or H_2_O_2_, through reduction of O_2_, using NADPH as the electron donor [110]. The complex is formed by a catalytic core located in the membrane and also called NOX, and structural and regulatory proteins located in the cytosol and in the membrane (Figure 3). The mammalian genome encodes seven homologous genes: NOX1, NOX2, NOX3, NOX4, NOX5, DUOX1, and DUOX2 [114,115] (Figure 3).

The first NOX isoform described, NOX2, was initially found in phagocytic cells, such as macrophages, eosinophils, and neutrophils [116], and is the most studied to date. The subunits located in the membrane are NOX2 (or gp91^phox^), which consists of the catalytic core of the enzyme, and p22^phox^, NOX2, and p22^phox^ subunits form the flavocytochrome b558. Additional subunits are recruited from the cytosol during complex activation. They include the proteins p67^phox^ (activator subunit), p47^phox^ (organizer subunit), and p40^phox^ (regulatory subunit, which helps p67^phox^ as activator subunit), as well as the small GTPases Rac 1 or 2 [110] (Figure 3). Of note, the existence of different subunits in different compartments assures that the enzyme will only be activated after a specific stimulus that induce cytosolic subunits migration to the membrane and their association with the membrane subunits.

Currently, it is known that NOX is ubiquitously expressed; however, its different isoforms are regulated and expressed in a tissue-specific manner. Our group was the first to describe the expression of some components of NOX2 in rat pancreatic β-cells [117] and in human β-cells [118]. In addition to NOX2, rodent and human pancreatic β-cells express NOX1 and NOX4 [117,118,119,120], and human β-cells express NOX5, absent in rodent β-cells [121]. We have also detected the expression of DUOX1 and DUOX2 in islets and rodent β-cell lines (unpublished data).

Our group and others have shown the participation of NOX in the physiology of insulin secretion. In this regard, NOX inhibition with a non-specific inhibitor (DPI) or with p47^phox^ oligonucleotide antisense caused a reduction of GSIS in islets [86], and NOX inhibition with Rac1 siRNA also led to the decrease of GSIS in INS 832/13 β-cells [122]. Furthermore, NOX inhibition with DPI inhibited palmitate-induced increase in O_2_^•−^ and insulin secretion in rat islets [123]. The crucial involvement of NOX4-derived H_2_O_2_ in the GSIS was recently shown and is reviewed elsewhere [88].

Despite its role in β-cell physiology, the sustained activity of NOX may contribute to the dysfunction of these cells associated with the development and progression of DM [124,125], with significant implications for the onset of metabolic dysfunction under stressful conditions [107,126]. In addition to its implication in DM, several NOX isoforms have been associated to the pathogenesis of a wide variety of diseases, such as cancer [127], hypertension [128,129], pulmonary fibrosis [130], kidney disease [131], and neurodegenerative diseases [114], among others.

The involvement of NOX in β-cell dysfunction and death under conditions that mimic T2D, such as exposure to high concentrations of glucose and saturated FAs, has been studied over the last decades. It has been shown that some NOX subunits have increased expression in animal models of T2D [132,133] and in diabetic human islets [133]. Although it seems clear that the expression of NOX is increased in β-cells under glucotoxic conditions in vitro [134,135,136], there are some conflicting results in the literature regarding the direct involvement of NOX in the glucotoxicity of these cells. INS-1 (rat β-cells) and 1.1B4 (human β-cells) acutely exposed to high glucose had activated Rac1, together with apoptosis and ROS production, all of which were attenuated by Rac1 specific inhibition [136]. In line with these, NIT-1 (mice β-cells) exposed to 48 h of high glucose concentration had increased apoptosis and NOX2 expression, in addition to decreased GSIS. This was rescued by NOX2 siRNA [134]. Taken together, these observations imply NOX2 as a culprit in glucotoxicity.

We have shown that islets from NOX2 KO mice cultured for 1–2 days in 10 mM of glucose, which is the standard glucose concentration in culture conditions, had increased GSIS compared to wild-type islets [137]. This result points to NOX2 as a negative modulator of GSIS, as also observed by Li and col [138]. However, after long-term (1 week) or prolonged (3 weeks) culture in 10 mM of glucose, there was no differences in GSIS patterns, and islets from both genotypes had a similar rate of glucose-induced apoptosis, and thus NOX2 might not be responsible for the glucotoxicity in β-cells [137].

Regarding the involvement of NOX in lipotoxicity in β-cells, our laboratory found that rat islets exposed to palmitate had increased protein expression of the NOX subunit p47^phox^, and increased NOX-derived O_2_^•−^, as this effect was reversed by NOX inhibition with DPI or p47^phox^ oligonucleotide antisense [139]. Using another non-specific NOX inhibitor (apocynin), the enzyme was shown to play a role in the dysfunction of insulin secretion caused by 24-h exposure to palmitate in BRIN-BD11 β-cell line and in mice islets [140]. In addition, we recently showed a relationship between the activation of NOX and the activation of GPR40 in BRIN-BD11 β-cells [141].

The specific role of NOX2 in lipotoxicity was demonstrated in NIT-1 β-cells, as the use of NOX2 siRNA protected against palmitate-induced dysfunction and apoptosis [142]. The specific role of NOX4 was also evidenced in high-fat diet-induced glucose intolerance in C57BL/6 mice using NOX4-selective inhibitor, GLX351322 [120]. Interestingly, a recent study used three inhibitors: ML171, Phox-I2, and GLX7013114, respectively, specific-inhibitors of NOX1, NOX2, and NOX4, in order to assess the impact of each isoform on the dysfunction of human islets and the human β-cell line EndoC-βH1 exposed to high glucose and palmitate [143]. They showed that NOX1 has no role in this scenario, while both NOX2 and NOX4 play a role in the glucolipotoxicity [143].

Confirming the role of NOX2 in the lipotoxicity in β-cells, using transgenic mice expressing a genetically-encoded H_2_O_2_ sensor, we recently demonstrated that palmitate leads to an early and transient H_2_O_2_ production via NOX2, which is mainly responsible for the early loss of β-cell function and viability [144]. Thus, NOX2 modulation could be a potential therapy against FA-induced β-cell dysfunction in obese and T2D patients.

Therefore, previous studies with inhibitors without specificity to the enzyme or to its different isoforms, such as DPI and apocynin, provided clues about the pathophysiological role of NOX. Furthermore, the development of several specific and selective inhibitors to different NOX isoforms has brought interesting results in recent years [145,146,147] and, thus, evidences indicate that NOX2/4 inhibition may represent a target for the preservation of β-cell function in the context of lipotoxicity during the development of T2D.

## 5. ER Stress in Lipotoxicity

ER stress has been implicated in lipotoxicity in pancreatic β-cells and in the development of diabetes [148,149,150,151]. The ER is an important organelle for lipid and protein biosynthesis and Ca^2+^ storage, with an important role in the capacity of secretory cells, such as β-cells, to adequately respond to increase in secretory demands [150,152]. During ER stress, characterized by accumulation of unfolded and misfolded proteins in the ER lumen, the capacity of the organelle to adequately fold proteins is disrupted. In order to restore the ER folding capacity, the unfolded protein response (UPR) is activated [148,149,150,152,153].

The UPR consists in three main signaling branches, initiated by sensor transmembrane proteins, namely: protein kinase RNA-like ER kinase (PERK), transcription factor 6 (ATF6), and inositol requiring ER-to nucleus kinase-like ER-associated kinase (IRE1). These three proteins remain inactivated when bonded to the ER chaperone immunoglobulin heavy-chain-binding protein (BiP), also known as glucose-regulated protein 78 (GRP78) or heat-shock protein A5 (HSPA5); however, accumulation of unfolded and misfolded proteins leads to BiP release from these transmembrane proteins, leading to activation of UPR [148,149,150,152,153,154]. Of note, there are some indications that unfolded proteins can interact directly with IRE1, and probably PERK, triggering their activation [155,156].

Activated PERK phosphorylates the α-subunit of the eukaryotic translational initiation factor 2 (eIF2α), which attenuates general protein translation, decreasing ER load, but at the same time facilitates translation of specific proteins, such as the transcription factor ATF4 [148,149,150,152,153,155]. ATF4 leads to expression of xbp1, gene involved in antioxidant response, and C/EBP homologous protein (CHOP) [148,149,150,152,153,155]. Together with CHOP, ATF4 upregulates expression of the DNA damage-inducible protein-34 (GADD34) [148,149,150,152,153], which is important to restore translational recovery, since it promotes eIF2α dephosphorylation [157,158]. Although this is an important function, prolonged CHOP expression on ER stress conditions is related to apoptosis induction [148,149,150,152,153].

Activated IRE1α alternatively splices the mRNA for X-box-binding protein 1 (XPB-1), which leads to translation of the XBP-1 transcription factor [148,149,150,152,153]. XBP-1 transcribes genes important for ER expansion, ER protein folding, and the ER-associated degradation (ERAD) pathway [148,155]. Another beneficial effect is due to its endonuclease activity, IRE1 promotes degradation of other mRNAs, attenuating ER load; however, it also leads to degradation of ER chaperones mRNAs, and during prolonged or stronger ER stress this contributes to cell death [150,155]. Of note, prolonged activation of this branch is also involved in activation of JNK and IRE1-dependent decay of mRNA (RIDD), which contribute to β-cell death during ER stress [159,160].

BiP-released ATF6 is transferred to the Golgi apparatus, via vesicular transport, where it is cleaved by Golgi-proteases, releasing the p50 fragment, which functions as a transcription factor [148,149,150,152,153]. ATF6 upregulates expression of ER chaperones and XBP-1 [161], showing a crosstalk with the IRE1 branch of the UPR. Of note, expression of IRE1 is also induced by PERK-ATF4 branch [162]. These crosstalks between the UPR branches seem to be important to modulate responses depending on the duration or intensity of the stress.

Thus, the combination of these pathways promotes decrease in the ER load by attenuation of protein translation, increase of misfolded protein degradation, ER folding capacity, ER size, and expression of ER chaperones. If homeostasis is achieved, the UPR sensors are inactivated, by BiP binding, and the cell continues to work properly. However, when homeostasis is not restored, keeping PERK and IRE1 branches activated, this will induce pro-apoptotic pathways, such as expression of CHOP, activation of JNK, inhibition of B-cell lymphoma 2 (Bcl-2) and activation of death protein 5 (DP5) and p53-upregulated modulator of apoptosis (PUMA) [149,155,163,164,165].

Due to their secretory capacity and adaptation to secrete high amount of insulin in response to increase in glucose concentration, pancreatic β-cells are especially sensitive to ER stress [148,149,150,152]. Protein accumulation in the ER lumen of β-cells can be induced by different mechanisms, such as diminished capacity to appropriately fold protein, increased demand for insulin production or disruption on ER-Golgi protein traffic [150]. One of the main causes for disruption of protein folding capacity is the decrease of ER Ca^2+^ concentration, since high concentration of this ion is necessary for ER-chaperones function. Disruption of the SERCA2 pump, by decreasing its expression or activity, is one of the main mechanisms by which cytokines and glucotoxicity act [150,166]. However, in the case of lipotoxicity, although decrease in ER Ca^2+^ is observed in β-cells exposed to specific FFAs [148,149,150], in animal models of obesity and T2D humans [150,167] there are still some controversies on how it is achieved, with some evidence pointing it as a consequence of the lipotoxicity-induced protein overload in the ER, rather than a direct effect [150,167,168]. Other studies, however, suggested a more direct effect of FFAs in the regulation of ER Ca^2+^ concentration. Of note, in non-β-cells the effect of the ER membrane lipid composition on SERCA2 function and ER stress induction was already shown [150,169]. In β-cells lipotoxicity-induced downregulation of sorcin, a Ca^2+^ sensor important for ER Ca^2+^ maintenance, may also be involved in this process [170]. Of note, overexpression of sorcin in β-cells improves glucose tolerance and β-cell function in mice exposed to a high-fat diet, increasing the ATF6 signaling [170].

The induction of oxidative stress by lipotoxicity is also involved in an ER-Ca^2+^ decrease in β-cells [104,152], and may involve ROS production by NOX and mitochondrial oxidative stress [152]. In addition, oxidative stress in the ER lumen is induced by oxidative unbalance during oxidative protein folding process, leading to increased formation of H_2_O_2._ The protein disulfide isomerase (PDI) promotes formation of disulfide bonds in ER proteins, being reduced in this process [78,152]. For a proper oxidative folding in the ER, the reduced PDI is oxidized by the ER oxidoreductase ER members (ERO1α and ERO1β), generating H_2_O_2_, which in normal conditions is rapidly inactivated by specific enzymes [78]. Thus, redox homeostasis is important to maintain ER folding capacity [78,152]. Overexpression of ERO1α is induced in β-cells exposed to palmitate [171], and hyperactivation of this enzyme can increase H_2_O_2_ formation and ER stress activation [172]. Indeed, accumulation of H_2_O_2_ in the ER lumen was shown to be necessary for FFA-induced ER stress in β-cells [173], linking oxidative stress to ER stress in lipotoxicity. This interconnection has important harmful consequences, since oxidative stress induced by lipotoxicity leads to ER-Ca^2+^ decrease, compromising ER folding capacity, which increases ROS production by ERO1α [152]. In addition, type I IP3 receptors (IP3R) are stimulated by ERO1α-produced ROS, which further contributes to ER Ca^2+^ release. In this scenario, overexpression of ERO1α is induced by both palmitate and prolonged ER stress-induced CHOP, in a positive feedback that amplifies and prolongs the ER stress [152].

Particularly for β-cells, in which pro-insulin production accounts for 50% of the total protein, this generates a delicate situation, since proper pro-insulin folding is dependent on oxidative folding mechanisms [78,152]. Thus, the oxidative stress leading to ER stress induced by FFA in β-cells may also be linked indirectly to the second cause for induction of protein accumulation in the ER, namely increased demand for insulin production. This would increase the production of H_2_O_2_, and it is suggested that ER has limited enzymatic antioxidant activity, being more susceptible to increases in protein folding demands [78,152]. In normal conditions β-cell can handle this increased demand [150], however exposure to some FFAs, such as palmitate, can interfere in redox and Ca^2+^ ER homeostasis, as described above, which will contribute to β-cell death, by inducing prolonged and strong ER stress.

A third mechanism that can induce ER protein accumulation is the disruption on ER-Golgi protein traffic [150]. Exposure of β-cells to FFAs, especially saturated, can disrupt ER-to-Golgi protein trafficking [150,168,174]. This effect may be due to the capacity of FFAs to modulate the expression of proteins involved in the transport of glycoproteins from ER-to-Golgi, such as lectin mannose-binding 1 and 2 (LMAN1 and LMAN2) [171]. FFAs can also downregulate expression of enzymes involved in pro-hormones processing, impairing insulin processing, which could lead to a disruption of protein trafficking and contribute to accumulation of proteins in the ER [150]. Modification of the ER membrane lipid composition is also suggested as a mechanism for lipotoxicity to disrupt ER-to-Golgi protein trafficking, influencing transport vesicle formation, and lipid rafts’ organization [150,175].

Induction of ER stress by FFAs is shown in different models of β-cell failure, implicating its role in the lipotoxic effects on β-cell function and death [148,149,150,152]. However, the use of specific types of FFAs in different models show that ER stress intensity and specific patterns of UPR branches activation will depend on the type or combination of different FFAs [77,149,151,176,177]. In general, saturated FFAs will lead to stronger ER stress activation compared to unsaturated ones [149,177]. Many studies use palmitate and oleate, as representant of saturated and unsaturated FFA, respectively, since they are the most abundant FFAs in humans [148,149]. Thus, palmitate leads to stronger activation of the PERK and IRE1 branches compared to oleate, with similar activation of ATF6 [151]. This translates to increased β-cell death marked by CHOP expression by palmitate, but not by oleate [149,151].

As discussed above, FFAs’ effects on ER stress induction may be direct or indirect, and involve not one, but many mechanisms that contribute to both straitening and prolonging the ER stress, compromising β-cell viability and function. It may involve oxidative stress in other cellular components and contribution of NOX [104,178,179,180], as further discussed in the last topic of this review. In addition, there are other factors that will contribute for the intensity of the lipotoxic effects in vivo, such as high glucose concentration, due to insulin resistance, as well as the inflammation increase caused by dyslipidemia [76,153], which may also influence ER stress in these cells. Taken together, induction of ER stress by lipotoxicity and the crosstalk with oxidative stress is an important feature observed both in vitro and in vivo, and may represent an important target for recovery of β-cell function during lipotoxic conditions.

## 6. NOX and ER Stress

As previously discussed, oxidative and ER stresses are highly interconnected biological processes that regulate a wide variety of signaling steps in the cell. The two cellular stresses profoundly impact normal physiology, as well as forming a vicious cycle in a wide variety of pathological conditions, including metabolic, neurodegenerative and inflammatory diseases [181]. Therefore, therapies that target both stresses may be more effective in treating these illnesses.

The association between ROS generation and ER stress has been studied in several cell types, including pancreatic β-cells. β-cells from CHOP-deficient diabetic mice were shown to be more resistant to oxidative stress [182] and in a phosphorylated eIf2α deficient mouse model, β-cells exhibited increased apoptosis and oxidative stress [183]. Regarding a crosstalk between NOX and ER stress, some examples in different tissues, including pancreatic islets, are shown in Table 1.

In cardiac cells of diabetic mice, NOX inhibition (with apocynin) also inhibited ER stress activation. A similar effect was found in Rac1 KO mice [184]. In macrophages, the induction of ER stress increases NOX expression. In contrast, NOX deletion in these cells decreases apoptosis and the expression of some markers of ER stress (ATF4 and CHOP) [185]. In neutrophils, NOX activation (with PMA) caused UPR activation via PERK and IRE-1 pathways, and NOX inhibition with DPI caused UPR attenuation [186]. In addition, it has been shown that NOXs affect ER Ca^2+^ receptors such as ryanodine receptors (RyR) and inositol triphosphate receptors (IP3R), and therefore lead to UPR activation by modulating ER Ca^2+^ levels [187,188].

Despite existing evidence in other cell types, the relationship between NOX and ER stress, leading to apoptosis and dysfunction of pancreatic β-cells, has been clarified only recently. We have shown that the transient increase in cytosolic H_2_O_2_ levels in islets exposed to palmitic acid coincides with the previously reported time-point of palmitate-induced UPR [144,151]. This suggests that NOX2-derived H_2_O_2_ could play a role in the activation of ER stress. However, when inhibiting NOX with a pharmacological inhibitor, VAS2870, we saw no protection from palmitate-induced activation of UPR response [144]. Using islets exposed to proinflammatory cytokines, we have recently proposed that NOX2-induced H_2_O_2_ production is ER stress dependent, as the phosphorylation of two important UPR proteins (p-eIF2-α and p-IRE1) precedes H_2_O_2_ production by NOX2 and that the attenuation of ER stress delays the cytokine-induced cytosolic H_2_O_2_ peak [189].

## 7. Concluding Remarks

**Figure 4:**
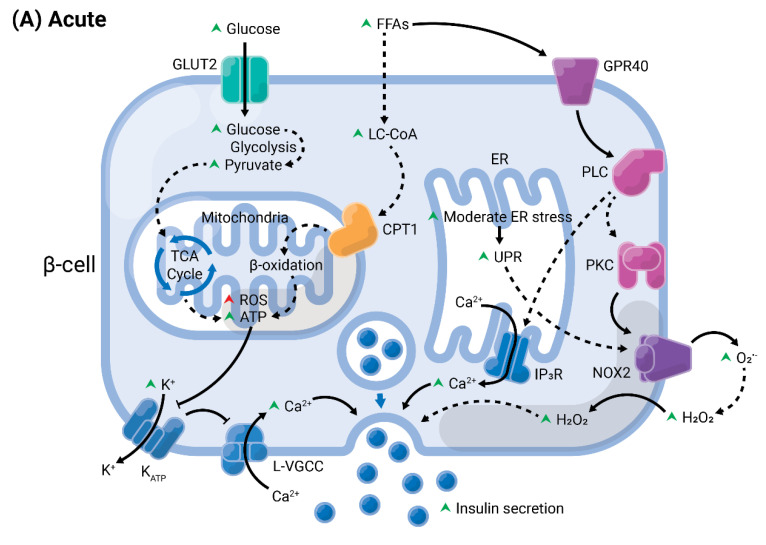

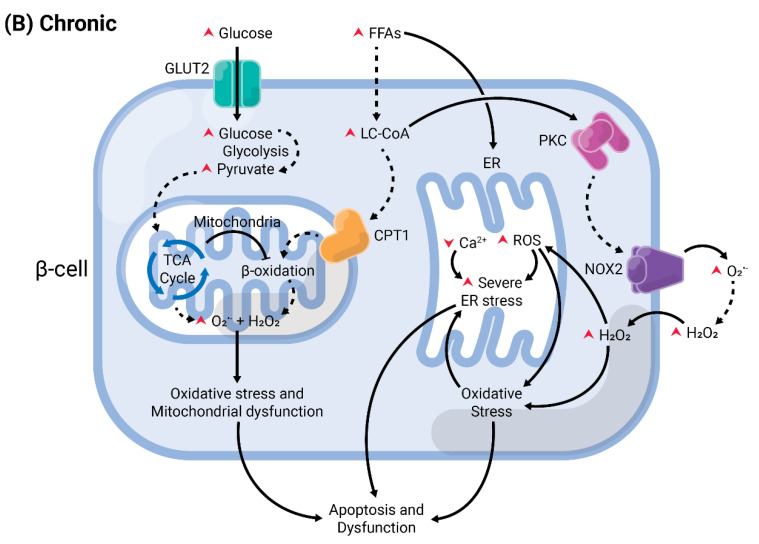
Summary of acute versus chronic effects of fatty acids (FAs) in pancreatic β-cells. Glucose enters the β-cell through specific transporters located at the plasma membrane, GLUT2 (in rodents). After its phosphorylation by glucokinase, it undergoes various modifications by enzymes from the glycolysis, until the generation of pyruvate. This enters the mitochondria and is oxidized in the tricarboxylic acid (TCA) cycle. The electrons are transferred to the electron transport chain, resulting in the generation of reactive oxygen species (ROS), as byproducts, and ATP. (**A**) In acute conditions, FAs enter the cells and are converted into long-chain fatty-acyl CoA (LC-CoA), which is translocated to the mitochondria via carnitine palmitoyltransferase 1 (CPT1) to be oxidized in the β-oxidation, generating ATP and ROS. The increase of ATP/ADP leads to the closure of K^+^ channels sensitive to ATP (K_ATP_) at the plasma membrane. The consequent membrane depolarization leads to the opening of L-type voltage-gated Ca^2+^ channels (L-VGCC). The rapid Ca^2+^ influx mobilizes insulin granules, which are released. Long-chain saturated or unsaturated FAs can also bind to Gαq-protein coupled receptor GPR40 at the plasma membrane. This activates the phospholipase C (PLC)/diacylglycerol (DAG) pathway, which respectively activates PKC and mobilizes Ca^2+^ from the endoplasmic reticulum (ER), potentiating GSIS. Activation of PKC may also activate NADPH oxidase 2 (NOX2) at the plasma to produce ROS, which are second messengers for GSIS. (**B**) Chronic exposure to FAs leads to depletion of ER Ca^2+^ and activation of ER stress, and potentiates ROS formation in all compartments (cytosol, mitochondria, and ER). Prolonged and unresolved oxidative stress, ER stress, and mitochondrial dysfunction culminate in apoptosis and dysfunction.

Obesity and related increase in FFA lead to diverse effects on pancreatic β-cells culminating in cell dysfunction, death and ultimately diabetes, as summarized in Figure 4. Among the several pathways, NADPH oxidase activation and ER stress are two important contributors for β-cells failure. Although very well studied individually, only recently the crosstalk between these apparently independent pathways has been suggested in pancreatic islets. However, the detailed mechanisms, hierarchy, and interplay is unknown. It will be very interesting to further investigate how each of these stresses contributes in activating and/or modulating each other and their potential for drug targeting.

## Figures and Tables

**Table 1:** Examples of studies involving NOX and ER stress crosstalk in different tissues.

NOX Influence on ER Stress	Model	Details	Ref
NOX inhibition → ER stress inhibition	Cardiomyocytes	NOX inhibition with apocynin or Rac1 KO mice	[184]
NOX inhibition → ER stress inhibition	Macrophages	NOX2 siRNA or NOX2 KO mice	[185]
NOX inhibition → ER stress inhibition	Neutrophils	HL60 cell line; NOX inhibition with DPI	[186]
NOX activation → ER stress activation	Neutrophils	HL60 cell line; NOX activation with PMA	[186]
NOX inhibition → no effect in ER stress	Mice islets	NOX inhibition with VAS2870	[144]
**ER Stress Influence on NOX**	**Model**	**Details**	**Ref**
ER stress induction → NOX increased expression	Macrophages	ER stress induction by cholesterol or 7-ketocholesterol	[185]
ER stress attenuation → NOX inhibition	Mice islets	ER stress attenuation with 4-PBA; NOX2-derived H_2_O_2_ peak was delayed	[189]

## Data Availability

Not applicable.

## References

[1] DeFronzo R.A., Ferrannini E., Groop L., Henry R.R., Herman W.H., Holst J.J., Hu F.B., Kahn C.R., Raz I., Shulman G.I. (2015). Type 2 diabetes mellitus. Nat. Rev. Dis. Primers.

[2] Defronzo R.A. (2009). Banting Lecture. From the triumvirate to the ominous octet: A new paradigm for the treatment of type 2 diabetes mellitus. Diabetes.

[3] Fuchsberger C., Flannick J., Teslovich T.M., Mahajan A., Agarwala V., Gaulton K.J., Ma C., Fontanillas P., Moutsianas L., McCarthy D.J. (2016). The genetic architecture of type 2 diabetes. Nature.

[4] Grarup N., Sandholt C.H., Hansen T., Pedersen O. (2014). Genetic susceptibility to type 2 diabetes and obesity: From genome-wide association studies to rare variants and beyond. Diabetologia.

[5] Meigs J.B., Cupples L.A., Wilson P.W. (2000). Parental transmission of type 2 diabetes: The Framingham Offspring Study. Diabetes.

[6] Weir G.C., Bonner-Weir S. (2004). Five stages of evolving beta-cell dysfunction during progression to diabetes. Diabetes.

[7] Cali A.M., Man C.D., Cobelli C., Dziura J., Seyal A., Shaw M., Allen K., Chen S., Caprio S. (2009). Primary defects in beta-cell function further exacerbated by worsening of insulin resistance mark the development of impaired glucose tolerance in obese adolescents. Diabetes Care.

[8] Lytrivi M., Castell A.L., Poitout V., Cnop M. (2020). Recent Insights Into Mechanisms of β-Cell Lipo- and Glucolipotoxicity in Type 2 Diabetes. J. Mol. Biol..

[9] Hasnain S.Z., Prins J.B., McGuckin M.A. (2016). Oxidative and endoplasmic reticulum stress in β-cell dysfunction in diabetes. J. Mol. Endocrinol..

[10] Burdge G.C., Calder P.C. (2015). Introduction to fatty acids and lipids. World Rev. Nutr. Diet..

[11] Hamilton J.A., Kamp F. (1999). How are free fatty acids transported in membranes? Is it by proteins or by free diffusion through the lipids?. Diabetes.

[12] Hao J.W., Wang J., Guo H., Zhao Y.Y., Sun H.H., Li Y.F., Lai X.Y., Zhao N., Wang X., Xie C. (2020). CD36 facilitates fatty acid uptake by dynamic palmitoylation-regulated endocytosis. Nat. Commun..

[13] Jay A.G., Simard J.R., Huang N., Hamilton J.A. (2020). SSO and other putative inhibitors of FA transport across membranes by CD36 disrupt intracellular metabolism, but do not affect FA translocation. J. Lipid. Res..

[14] Czech M.P. (2017). Insulin action and resistance in obesity and type 2 diabetes. Nat. Med..

[15] Montgomery M.K., Turner N. (2015). Mitochondrial dysfunction and insulin resistance: An update. Endocr. Connect..

[16] Petersen K.F., Befroy D., Dufour S., Dziura J., Ariyan C., Rothman D.L., DiPietro L., Cline G.W., Shulman G.I. (2003). Mitochondrial dysfunction in the elderly: Possible role in insulin resistance. Science.

[17] Bays H., Mandarino L., DeFronzo R.A. (2004). Role of the adipocyte, free fatty acids, and ectopic fat in pathogenesis of type 2 diabetes mellitus: Peroxisomal proliferator-activated receptor agonists provide a rational therapeutic approach. J. Clin. Endocrinol. Metab..

[18] Bays H.E., González-Campoy J.M., Bray G.A., Kitabchi A.E., Bergman D.A., Schorr A.B., Rodbard H.W., Henry R.R. (2008). Pathogenic potential of adipose tissue and metabolic consequences of adipocyte hypertrophy and increased visceral adiposity. Expert. Rev. Cardiovasc. Ther..

[19] Haber E.P., Ximenes H.M., Procópio J., Carvalho C.R., Curi R., Carpinelli A.R. (2003). Pleiotropic effects of fatty acids on pancreatic beta-cells. J. Cell Physiol..

[20] Haber E.P., Procópio J., Carvalho C.R., Carpinelli A.R., Newsholme P., Curi R. (2006). New insights into fatty acid modulation of pancreatic beta-cell function. Int. Rev. Cytol..

[21] Bermudez B., Ortega-Gomez A., Varela L.M., Villar J., Abia R., Muriana F.J., Lopez S. (2014). Clustering effects on postprandial insulin secretion and sensitivity in response to meals with different fatty acid compositions. Food Funct..

[22] Ježek P., Jabůrek M., Holendová B., Plecitá-Hlavatá L. (2018). Fatty Acid-Stimulated Insulin Secretion vs. Lipotoxicity. Molecules.

[23] Kunau W.H., Dommes V., Schulz H. (1995). beta-oxidation of fatty acids in mitochondria, peroxisomes, and bacteria: A century of continued progress. Prog. Lipid. Res..

[24] Reddy J.K., Hashimoto T. (2001). Peroxisomal beta-oxidation and peroxisome proliferator-activated receptor alpha: An adaptive metabolic system. Annu. Rev. Nutr..

[25] Itoh Y., Kawamata Y., Harada M., Kobayashi M., Fujii R., Fukusumi S., Ogi K., Hosoya M., Tanaka Y., Uejima H. (2003). Free fatty acids regulate insulin secretion from pancreatic beta cells through GPR40. Nature.

[26] Itoh Y., Hinuma S. (2005). GPR40, a free fatty acid receptor on pancreatic beta cells, regulates insulin secretion. Hepatol. Res..

[27] Gravena C., Mathias P.C., Ashcroft S.J. (2002). Acute effects of fatty acids on insulin secretion from rat and human islets of Langerhans. J. Endocrinol..

[28] Carpinelli A.R., Picinato M.C., Stevanato E., Oliveira H.R., Curi R. (2002). Insulin secretion induced by palmitate--a process fully dependent on glucose concentration. Diabetes Metab..

[29] Deeney J.T., Gromada J., Høy M., Olsen H.L., Rhodes C.J., Prentki M., Berggren P.O., Corkey B.E. (2000). Acute stimulation with long chain acyl-CoA enhances exocytosis in insulin-secreting cells (HIT T-15 and NMRI beta-cells). J. Biol. Chem..

[30] Stein D.T., Esser V., Stevenson B.E., Lane K.E., Whiteside J.H., Daniels M.B., Chen S., McGarry J.D. (1996). Essentiality of circulating fatty acids for glucose-stimulated insulin secretion in the fasted rat. J. Clin. investig..

[31] Stein D.T., Stevenson B.E., Chester M.W., Basit M., Daniels M.B., Turley S.D., McGarry J.D. (1997). The insulinotropic potency of fatty acids is influenced profoundly by their chain length and degree of saturation. J. Clin. Investig..

[32] Briscoe C.P., Tadayyon M., Andrews J.L., Benson W.G., Chambers J.K., Eilert M.M., Ellis C., Elshourbagy N.A., Goetz A.S., Minnick D.T. (2003). The orphan G protein-coupled receptor GPR40 is activated by medium and long chain fatty acids. J. Biol. Chem..

[33] Hirasawa A., Tsumaya K., Awaji T., Katsuma S., Adachi T., Yamada M., Sugimoto Y., Miyazaki S., Tsujimoto G. (2005). Free fatty acids regulate gut incretin glucagon-like peptide-1 secretion through GPR120. Nat. Med..

[34] Shapiro H., Shachar S., Sekler I., Hershfinkel M., Walker M.D. (2005). Role of GPR40 in fatty acid action on the beta cell line INS-1E. Biochem. Biophys Res. Commun..

[35] Kotarsky K., Nilsson N.E., Flodgren E., Owman C., Olde B. (2003). A human cell surface receptor activated by free fatty acids and thiazolidinedione drugs. Biochem. Biophys. Res. Commun..

[36] Poitout V. (2003). The ins and outs of fatty acids on the pancreatic beta cell. Trends Endocrinol. Metab..

[37] Gromada J. (2006). The free fatty acid receptor GPR40 generates excitement in pancreatic beta-cells. Endocrinology.

[38] Nolan C.J., Madiraju M.S., Delghingaro-Augusto V., Peyot M.L., Prentki M. (2006). Fatty acid signaling in the beta-cell and insulin secretion. Diabetes.

[39] Kebede M.A., Alquier T., Latour M.G., Poitout V. (2009). Lipid receptors and islet function: Therapeutic implications?. Diabetes Obes. Metab..

[40] Del Guerra S., Bugliani M., D’Aleo V., Del Prato S., Boggi U., Mosca F., Filipponi F., Lupi R. (2010). G-protein-coupled receptor 40 (GPR40) expression and its regulation in human pancreatic islets: The role of type 2 diabetes and fatty acids. Nutr. Metab. Cardiovasc. Dis..

[41] Vettor R., Granzotto M., De Stefani D., Trevellin E., Rossato M., Farina M.G., Milan G., Pilon C., Nigro A., Federspil G. (2008). Loss-of-function mutation of the GPR40 gene associates with abnormal stimulated insulin secretion by acting on intracellular calcium mobilization. J. Clin. Endocrinol. Metab..

[42] Garrido D.M., Corbett D.F., Dwornik K.A., Goetz A.S., Littleton T.R., McKeown S.C., Mills W.Y., Smalley T.L., Briscoe C.P., Peat A.J. (2006). Synthesis and activity of small molecule GPR40 agonists. Bioorg. Med. Chem. Lett..

[43] Bharate S.B., Rodge A., Joshi R.K., Kaur J., Srinivasan S., Kumar S.S., Kulkarni-Almeida A., Balachandran S., Balakrishnan A., Vishwakarma R.A. (2008). Discovery of diacylphloroglucinols as a new class of GPR40 (FFAR1) agonists. Bioorg. Med. Chem. Lett..

[44] Christiansen E., Urban C., Merten N., Liebscher K., Karlsen K.K., Hamacher A., Spinrath A., Bond A.D., Drewke C., Ullrich S. (2008). Discovery of potent and selective agonists for the free fatty acid receptor 1 (FFA(1)/GPR40), a potential target for the treatment of type II diabetes. J. Med. Chem..

[45] Negoro N., Sasaki S., Mikami S., Ito M., Suzuki M., Tsujihata Y., Ito R., Harada A., Takeuchi K., Suzuki N. (2010). Discovery of TAK-875: A Potent, Selective, and Orally Bioavailable GPR40 Agonist. ACS Med. Chem. Lett..

[46] Houze J.B., Zhu L., Sun Y., Akerman M., Qiu W., Zhang A.J., Sharma R., Schmitt M., Wang Y., Liu J. (2012). AMG 837: A potent, orally bioavailable GPR40 agonist. Bioorg. Med. Chem. Lett..

[47] Araki T., Hirayama M., Hiroi S., Kaku K. (2012). GPR40-induced insulin secretion by the novel agonist TAK-875: First clinical findings in patients with type 2 diabetes. Diabetes Obes. Metab..

[48] Vilas-Boas E.A., Karabacz N., Marsiglio-Librais G.N., Valle M.M.R., Nalbach L., Ampofo E., Morgan B., Carpinelli A.R., Roma L.P. (2020). Chronic activation of GPR40 does not negatively impact upon BRIN-BD11 pancreatic β-cell physiology and function. Pharmacol. Rep..

[49] Carlsson M., Wessman Y., Almgren P., Groop L. (2000). High levels of nonesterified fatty acids are associated with increased familial risk of cardiovascular disease. Arterioscler Thromb. Vasc. Biol..

[50] Salgin B., Ong K.K., Thankamony A., Emmett P., Wareham N.J., Dunger D.B. (2012). Higher fasting plasma free fatty acid levels are associated with lower insulin secretion in children and adults and a higher incidence of type 2 diabetes. J. Clin. Endocrinol. Metab..

[51] Mahendran Y., Cederberg H., Vangipurapu J., Kangas A.J., Soininen P., Kuusisto J., Uusitupa M., Ala-Korpela M., Laakso M. (2013). Glycerol and fatty acids in serum predict the development of hyperglycemia and type 2 diabetes in Finnish men. Diabetes Care.

[52] Risérus U., Willett W.C., Hu F.B. (2009). Dietary fats and prevention of type 2 diabetes. Prog. Lipid Res..

[53] Hodge A.M., English D.R., O’Dea K., Sinclair A.J., Makrides M., Gibson R.A., Giles G.G. (2007). Plasma phospholipid and dietary fatty acids as predictors of type 2 diabetes: Interpreting the role of linoleic acid. Am. J. Clin. Nutr..

[54] Sobczak A.I.S., Blindauer C.A., Stewart A.J. (2019). Changes in Plasma Free Fatty Acids Associated with Type-2 Diabetes. Nutrients.

[55] Poitout V., Amyot J., Semache M., Zarrouki B., Hagman D., Fontés G. (2010). Glucolipotoxicity of the pancreatic beta cell. Biochim. Biophys. Acta.

[56] Poitout V., Robertson R.P. (2008). Glucolipotoxicity: Fuel excess and beta-cell dysfunction. Endocr. Rev..

[57] Prentki M., Nolan C.J. (2006). Islet beta cell failure in type 2 diabetes. J. Clin. Investig..

[58] Poitout V., Hagman D., Stein R., Artner I., Robertson R.P., Harmon J.S. (2006). Regulation of the insulin gene by glucose and fatty acids. J. Nutr..

[59] Ritz-Laser B., Meda P., Constant I., Klages N., Charollais A., Morales A., Magnan C., Ktorza A., Philippe J. (1999). Glucose-induced preproinsulin gene expression is inhibited by the free fatty acid palmitate. Endocrinology.

[60] Hagman D.K., Hays L.B., Parazzoli S.D., Poitout V. (2005). Palmitate inhibits insulin gene expression by altering PDX-1 nuclear localization and reducing MafA expression in isolated rat islets of Langerhans. J. Biol. Chem..

[61] Wang Y., Wang P.Y., Takashi K. (2006). Chronic effects of different non-esterified fatty acids on pancreatic islets of rats. Endocrine.

[62] Maris M., Robert S., Waelkens E., Derua R., Hernangomez M.H., D’Hertog W., Cnop M., Mathieu C., Overbergh L. (2013). Role of the saturated nonesterified Fatty Acid palmitate in Beta cell dysfunction. J. Proteome Res..

[63] Dixon G., Nolan J., McClenaghan N.H., Flatt P.R., Newsholme P. (2004). Arachidonic acid, palmitic acid and glucose are important for the modulation of clonal pancreatic beta-cell insulin secretion, growth and functional integrity. Clin. Sci..

[64] El-Assaad W., Buteau J., Peyot M.L., Nolan C., Roduit R., Hardy S., Joly E., Dbaibo G., Rosenberg L., Prentki M. (2003). Saturated fatty acids synergize with elevated glucose to cause pancreatic beta-cell death. Endocrinology.

[65] Oh Y.S., Bae G.D., Baek D.J., Park E.Y., Jun H.S. (2018). Fatty Acid-Induced Lipotoxicity in Pancreatic Beta-Cells During Development of Type 2 Diabetes. Front. Endocrinol..

[66] Keane D.C., Takahashi H.K., Dhayal S., Morgan N.G., Curi R., Newsholme P. (2011). Arachidonic acid actions on functional integrity and attenuation of the negative effects of palmitic acid in a clonal pancreatic β-cell line. Clin. Sci..

[67] Maedler K., Oberholzer J., Bucher P., Spinas G.A., Donath M.Y. (2003). Monounsaturated fatty acids prevent the deleterious effects of palmitate and high glucose on human pancreatic beta-cell turnover and function. Diabetes.

[68] Eitel K., Staiger H., Brendel M.D., Brandhorst D., Bretzel R.G., Häring H.U., Kellerer M. (2002). Different role of saturated and unsaturated fatty acids in beta-cell apoptosis. Biochem Biophys Res. Commun..

[69] Keane D., Newsholme P. (2008). Saturated and unsaturated (including arachidonic acid) non-esterified fatty acid modulation of insulin secretion from pancreatic beta-cells. Biochem. Soc. Trans..

[70] Fürstova V., Kopska T., James R.F., Kovar J. (2008). Comparison of the effect of individual saturated and unsaturated fatty acids on cell growth and death induction in the human pancreatic beta-cell line NES2Y. Life Sci..

[71] Dhayal S., Welters H.J., Morgan N.G. (2008). Structural requirements for the cytoprotective actions of mono-unsaturated fatty acids in the pancreatic beta-cell line, BRIN-BD11. Br. J. Pharmacol..

[72] Kato T., Shimano H., Yamamoto T., Ishikawa M., Kumadaki S., Matsuzaka T., Nakagawa Y., Yahagi N., Nakakuki M., Hasty A.H. (2008). Palmitate impairs and eicosapentaenoate restores insulin secretion through regulation of SREBP-1c in pancreatic islets. Diabetes.

[73] Welters H.J., Tadayyon M., Scarpello J.H., Smith S.A., Morgan N.G. (2004). Mono-unsaturated fatty acids protect against beta-cell apoptosis induced by saturated fatty acids, serum withdrawal or cytokine exposure. FEBS Lett..

[74] Eizirik D.L., Pasquali L., Cnop M. (2020). Pancreatic β-cells in type 1 and type 2 diabetes mellitus: Different pathways to failure. Nat. Rev. Endocrinol..

[75] Rahier J., Guiot Y., Goebbels R.M., Sempoux C., Henquin J.C. (2008). Pancreatic beta-cell mass in European subjects with type 2 diabetes. Diabetes Obes. Metab..

[76] Cnop M., Welsh N., Jonas J.C., Jörns A., Lenzen S., Eizirik D.L. (2005). Mechanisms of pancreatic beta-cell death in type 1 and type 2 diabetes: Many differences, few similarities. Diabetes.

[77] Plötz T., von Hanstein A.S., Krümmel B., Laporte A., Mehmeti I., Lenzen S. (2019). Structure-toxicity relationships of saturated and unsaturated free fatty acids for elucidating the lipotoxic effects in human EndoC-βH1 beta-cells. Biochim. Biophys. Acta Mol. Basis Dis..

[78] Lenzen S. (2017). Chemistry and biology of reactive species with special reference to the antioxidative defence status in pancreatic β-cells. Biochim. Biophys. Acta Gen. Subj..

[79] Fang F.C. (2011). Antimicrobial actions of reactive oxygen species. mBio.

[80] Herb M., Schramm M. (2021). Functions of ROS in Macrophages and Antimicrobial Immunity. Antioxidants.

[81] Babior B.M., Kipnes R.S., Curnutte J.T. (1973). Biological defense mechanisms. The production by leukocytes of superoxide, a potential bactericidal agent. J. Clin. Investig..

[82] Sies H., Jones D.P. (2020). Reactive oxygen species (ROS) as pleiotropic physiological signalling agents. Nat. Rev. Mol. Cell Biol..

[83] Reczek C.R., Chandel N.S. (2015). ROS-dependent signal transduction. Curr. Opin. Cell Biol..

[84] Weidinger A., Kozlov A.V. (2015). Biological Activities of Reactive Oxygen and Nitrogen Species: Oxidative Stress versus Signal Transduction. Biomolecules.

[85] Pi J., Bai Y., Zhang Q., Wong V., Floering L.M., Daniel K., Reece J.M., Deeney J.T., Andersen M.E., Corkey B.E. (2007). Reactive oxygen species as a signal in glucose-stimulated insulin secretion. Diabetes.

[86] Morgan D., Rebelato E., Abdulkader F., Graciano M.F., Oliveira-Emilio H.R., Hirata A.E., Rocha M.S., Bordin S., Curi R., Carpinelli A.R. (2009). Association of NAD(P)H oxidase with glucose-induced insulin secretion by pancreatic beta-cells. Endocrinology.

[87] Ježek P., Holendová B., Jabůrek M., Tauber J., Dlasková A., Plecitá-Hlavatá L. (2021). The Pancreatic β-Cell: The Perfect Redox System. Antioxidants.

[88] Plecitá-Hlavatá L., Jabůrek M., Holendová B., Tauber J., Pavluch V., Berková Z., Cahová M., Schröder K., Brandes R.P., Siemen D. (2020). Glucose-Stimulated Insulin Secretion Fundamentally Requires H. Diabetes.

[89] Sies H. (2017). Hydrogen peroxide as a central redox signaling molecule in physiological oxidative stress: Oxidative eustress. Redox Biol..

[90] Lakey J.R., Suarez-Pinzon W.L., Strynadka K., Korbutt G.S., Rajotte R.V., Mabley J.G., Szabó C., Rabinovitch A. (2001). Peroxynitrite is a mediator of cytokine-induced destruction of human pancreatic islet beta cells. Lab. investig..

[91] McCord J.M., Fridovich I. (1969). Superoxide dismutase. An enzymic function for erythrocuprein (hemocuprein). J. Biol. Chem..

[92] Kaneto H., Matsuoka T.A., Nakatani Y., Kawamori D., Miyatsuka T., Matsuhisa M., Yamasaki Y. (2005). Oxidative stress, ER stress, and the JNK pathway in type 2 diabetes. J. Mol. Med..

[93] Robertson R.P. (2004). Chronic oxidative stress as a central mechanism for glucose toxicity in pancreatic islet beta cells in diabetes. J. Biol. Chem..

[94] Lenzen S., Drinkgern J., Tiedge M. (1996). Low antioxidant enzyme gene expression in pancreatic islets compared with various other mouse tissues. Free Radic. Biol. Med..

[95] Tiedge M., Lortz S., Drinkgern J., Lenzen S. (1997). Relation between antioxidant enzyme gene expression and antioxidative defense status of insulin-producing cells. Diabetes.

[96] Lenzen S. (2008). Oxidative stress: The vulnerable beta-cell. Biochem. Soc. Trans..

[97] Grankvist K., Marklund S.L., Täljedal I.B. (1981). CuZn-superoxide dismutase, Mn-superoxide dismutase, catalase and glutathione peroxidase in pancreatic islets and other tissues in the mouse. Biochem. J..

[98] Godoy J.R., Funke M., Ackermann W., Haunhorst P., Oesteritz S., Capani F., Elsässer H.P., Lillig C.H. (2011). Redox atlas of the mouse. Immunohistochemical detection of glutaredoxin-, peroxiredoxin-, and thioredoxin-family proteins in various tissues of the laboratory mouse. Biochim. Biophys. Acta.

[99] Stancill J.S., Broniowska K.A., Oleson B.J., Naatz A., Corbett J.A. (2019). Pancreatic β-cells detoxify H. J. Biol. Chem..

[100] Benáková Š., Holendová B., Plecitá-Hlavatá L. (2021). Redox Homeostasis in Pancreatic β-Cells: From Development to Failure. Antioxidants.

[101] Fu J., Cui Q., Yang B., Hou Y., Wang H., Xu Y., Wang D., Zhang Q., Pi J. (2017). The impairment of glucose-stimulated insulin secretion in pancreatic β-cells caused by prolonged glucotoxicity and lipotoxicity is associated with elevated adaptive antioxidant response. Food Chem. Toxicol..

[102] Diaz-Ganete A., Quiroga-de-Castro A., Mateos R.M., Medina F., Segundo C., Lechuga-Sancho A.M. (2021). Toxicity Induced by Cytokines, Glucose, and Lipids Increase Apoptosis and Hamper Insulin Secretion in the 1.1E7 Beta Cell-Line. Int. J. Mol. Sci..

[103] Oprescu A.I., Bikopoulos G., Naassan A., Allister E.M., Tang C., Park E., Uchino H., Lewis G.F., Fantus I.G., Rozakis-Adcock M. (2007). Free fatty acid-induced reduction in glucose-stimulated insulin secretion: Evidence for a role of oxidative stress in vitro and in vivo. Diabetes.

[104] Ly L.D., Xu S., Choi S.K., Ha C.M., Thoudam T., Cha S.K., Wiederkehr A., Wollheim C.B., Lee I.K., Park K.S. (2017). Oxidative stress and calcium dysregulation by palmitate in type 2 diabetes. Exp. Mol. Med..

[105] Oguntibeju O.O. (2019). Type 2 diabetes mellitus, oxidative stress and inflammation: Examining the links. Int. J. Physiol. Pathophysiol. Pharmacol..

[106] Newsholme P., Keane K.N., Carlessi R., Cruzat V. (2019). Oxidative stress pathways in pancreatic β-cells and insulin-sensitive cells and tissues: Importance to cell metabolism, function, and dysfunction. Am. J. Physiol. Cell Physiol..

[107] Piro S., Anello M., Di Pietro C., Lizzio M.N., Patanè G., Rabuazzo A.M., Vigneri R., Purrello M., Purrello F. (2002). Chronic exposure to free fatty acids or high glucose induces apoptosis in rat pancreatic islets: Possible role of oxidative stress. Metabolism.

[108] McNally J.S., Davis M.E., Giddens D.P., Saha A., Hwang J., Dikalov S., Jo H., Harrison D.G. (2003). Role of xanthine oxidoreductase and NAD(P)H oxidase in endothelial superoxide production in response to oscillatory shear stress. Am. J. Physiol. Heart Circ. Physiol..

[109] Fleming I., Michaelis U.R., Bredenkötter D., Fisslthaler B., Dehghani F., Brandes R.P., Busse R. (2001). Endothelium-derived hyperpolarizing factor synthase (Cytochrome P450 2C9) is a functionally significant source of reactive oxygen species in coronary arteries. Circ. Res..

[110] Babior B.M. (1999). NADPH oxidase: An update. Blood.

[111] Mehmeti I., Lortz S., Lenzen S. (2012). The H_2_O_2_-sensitive HyPer protein targeted to the endoplasmic reticulum as a mirror of the oxidizing thiol-disulfide milieu. Free Radic. Biol. Med..

[112] Sandalio L.M., Rodríguez-Serrano M., Romero-Puertas M.C., del Río L.A. (2013). Role of peroxisomes as a source of reactive oxygen species (ROS) signaling molecules. Subcell Biochem..

[113] Zorov D.B., Juhaszova M., Sollott S.J. (2014). Mitochondrial reactive oxygen species (ROS) and ROS-induced ROS release. Physiol. Rev..

[114] Bedard K., Krause K.H. (2007). The NOX family of ROS-generating NADPH oxidases: Physiology and pathophysiology. Physiol. Rev..

[115] Buvelot H., Jaquet V., Krause K.H. (2019). Mammalian NADPH Oxidases. Methods Mol. Biol.

[116] Berton G., Castaldi M.A., Cassatella M.A., Nauseef W.M. (2015). Editorial: Celebrating the 50th anniversary of the seminal discovery that the phagocyte respiratory burst enzyme is an NADPH oxidase. J. Leukoc. Biol..

[117] Oliveira H.R., Verlengia R., Carvalho C.R., Britto L.R., Curi R., Carpinelli A.R. (2003). Pancreatic beta-cells express phagocyte-like NAD(P)H oxidase. Diabetes.

[118] Rebelato E., Mares-Guia T.R., Graciano M.F., Labriola L., Britto L.R., Garay-Malpartida H.M., Curi R., Sogayar M.C., Carpinelli A.R. (2012). Expression of NADPH oxidase in human pancreatic islets. Life Sci..

[119] Uchizono Y., Takeya R., Iwase M., Sasaki N., Oku M., Imoto H., Iida M., Sumimoto H. (2006). Expression of isoforms of NADPH oxidase components in rat pancreatic islets. Life Sci..

[120] Anvari E., Wikström P., Walum E., Welsh N. (2015). The novel NADPH oxidase 4 inhibitor GLX351322 counteracts glucose intolerance in high-fat diet-treated C57BL/6 mice. Free Radic. Res..

[121] Bouzakri K., Veyrat-Durebex C., Holterman C., Arous C., Barbieux C., Bosco D., Altirriba J., Alibashe M., Tournier B.B., Gunton J.E. (2020). Beta-Cell-Specific Expression of Nicotinamide Adenine Dinucleotide Phosphate Oxidase 5 Aggravates High-Fat Diet-Induced Impairment of Islet Insulin Secretion in Mice. Antioxid Redox Signal.

[122] Kowluru A. (2011). Friendly, and not so friendly, roles of Rac1 in islet β-cell function: Lessons learnt from pharmacological and molecular biological approaches. Biochem. Pharmacol..

[123] Graciano M.F., Santos L.R., Curi R., Carpinelli A.R. (2011). NAD(P)H oxidase participates in the palmitate-induced superoxide production and insulin secretion by rat pancreatic islets. J. Cell Physiol..

[124] Taylor-Fishwick D.A. (2013). NOX, NOX Who is There? The Contribution of NADPH Oxidase One to Beta Cell Dysfunction. Front. Endocrinol..

[125] Sedeek M., Montezano A.C., Hebert R.L., Gray S.P., Di Marco E., Jha J.C., Cooper M.E., Jandeleit-Dahm K., Schiffrin E.L., Wilkinson-Berka J.L. (2012). Oxidative stress, Nox isoforms and complications of diabetes—Potential targets for novel therapies. J. Cardiovasc. Transl. Res..

[126] Newsholme P., Keane D., Welters H.J., Morgan N.G. (2007). Life and death decisions of the pancreatic beta-cell: The role of fatty acids. Clin. Sci..

[127] Blanchetot C., Boonstra J. (2008). The ROS-NOX connection in cancer and angiogenesis. Crit. Rev. Eukaryot. Gene Expr..

[128] Santos C.X., Nabeebaccus A.A., Shah A.M., Camargo L.L., Filho S.V., Lopes L.R. (2014). Endoplasmic reticulum stress and Nox-mediated reactive oxygen species signaling in the peripheral vasculature: Potential role in hypertension. Antioxid. Redox Signal.

[129] Sedeek M., Hébert R.L., Kennedy C.R., Burns K.D., Touyz R.M. (2009). Molecular mechanisms of hypertension: Role of Nox family NADPH oxidases. Curr. Opin. Nephrol. Hypertens.

[130] Hecker L., Cheng J., Thannickal V.J. (2012). Targeting NOX enzymes in pulmonary fibrosis. Cell Mol. Life Sci..

[131] You Y.H., Okada S., Ly S., Jandeleit-Dahm K., Barit D., Namikoshi T., Sharma K. (2013). Role of Nox2 in diabetic kidney disease. Am. J. Physiol. Renal. Physiol..

[132] Nakayama M., Inoguchi T., Sonta T., Maeda Y., Sasaki S., Sawada F., Tsubouchi H., Sonoda N., Kobayashi K., Sumimoto H. (2005). Increased expression of NAD(P)H oxidase in islets of animal models of Type 2 diabetes and its improvement by an AT1 receptor antagonist. Biochem. Biophys. Res. Commun..

[133] Syed I., Kyathanahalli C.N., Jayaram B., Govind S., Rhodes C.J., Kowluru R.A., Kowluru A. (2011). Increased phagocyte-like NADPH oxidase and ROS generation in type 2 diabetic ZDF rat and human islets: Role of Rac1-JNK1/2 signaling pathway in mitochondrial dysregulation in the diabetic islet. Diabetes.

[134] Yuan H., Lu Y., Huang X., He Q., Man Y., Zhou Y., Wang S., Li J. (2010). Suppression of NADPH oxidase 2 substantially restores glucose-induced dysfunction of pancreatic NIT-1 cells. FEBS J..

[135] Mohammed A.M., Kowluru A. (2013). Activation of apocynin-sensitive NADPH oxidase (Nox2) activity in INS-1 832/13 cells under glucotoxic conditions. Islets.

[136] Elumalai S., Karunakaran U., Lee I.K., Moon J.S., Won K.C. (2017). Rac1-NADPH oxidase signaling promotes CD36 activation under glucotoxic conditions in pancreatic beta cells. Redox Biol..

[137] de Souza A.H., Santos L.R., Roma L.P., Bensellam M., Carpinelli A.R., Jonas J.C. (2017). NADPH oxidase-2 does not contribute to β-cell glucotoxicity in cultured pancreatic islets from C57BL/6J mice. Mol. Cell Endocrinol..

[138] Li N., Li B., Brun T., Deffert-Delbouille C., Mahiout Z., Daali Y., Ma X.J., Krause K.H., Maechler P. (2012). NADPH oxidase NOX2 defines a new antagonistic role for reactive oxygen species and cAMP/PKA in the regulation of insulin secretion. Diabetes.

[139] Morgan D., Oliveira-Emilio H.R., Keane D., Hirata A.E., Santos da Rocha M., Bordin S., Curi R., Newsholme P., Carpinelli A.R. (2007). Glucose, palmitate and pro-inflammatory cytokines modulate production and activity of a phagocyte-like NADPH oxidase in rat pancreatic islets and a clonal beta cell line. Diabetologia.

[140] Michalska M., Wolf G., Walther R., Newsholme P. (2010). Effects of pharmacological inhibition of NADPH oxidase or iNOS on pro-inflammatory cytokine, palmitic acid or H_2_O_2_-induced mouse islet or clonal pancreatic β-cell dysfunction. Biosci. Rep..

[141] Nunes Marsiglio-Librais G., Aparecida Vilas-Boas E., Carlein C., Hoffmann M.D.A., Roma L.P., Carpinelli A.R. (2020). Evidence for NADPH oxidase activation by GPR40 in pancreatic β-cells. Redox Rep..

[142] Yuan H., Zhang X., Huang X., Lu Y., Tang W., Man Y., Wang S., Xi J., Li J. (2010). NADPH oxidase 2-derived reactive oxygen species mediate FFAs-induced dysfunction and apoptosis of β-cells via JNK, p38 MAPK and p53 pathways. PLoS ONE.

[143] Wang X., Elksnis A., Wikström P., Walum E., Welsh N., Carlsson P.O. (2018). The novel NADPH oxidase 4 selective inhibitor GLX7013114 counteracts human islet cell death in vitro. PLoS ONE.

[144] Vilas-Boas E.A., Nalbach L., Ampofo E., Lucena C.F., Naudet L., Ortis F., Carpinelli A.R., Morgan B., Roma L.P. (2020). Transient NADPH oxidase 2-dependent H_2_O_2_ production drives early palmitate-induced lipotoxicity in pancreatic islets. Free Radic. Biol. Med..

[145] Kleniewska P., Piechota A., Skibska B., Gorąca A. (2012). The NADPH oxidase family and its inhibitors. Arch. Immunol. Ther. Exp..

[146] Cifuentes-Pagano E., Meijles D.N., Pagano P.J. (2014). The quest for selective nox inhibitors and therapeutics: Challenges, triumphs and pitfalls. Antioxid. Redox Signal.

[147] Altenhöfer S., Radermacher K.A., Kleikers P.W., Wingler K., Schmidt H.H. (2015). Evolution of NADPH Oxidase Inhibitors: Selectivity and Mechanisms for Target Engagement. Antioxid. Redox Signal.

[148] Cnop M., Igoillo-Esteve M., Cunha D.A., Ladrière L., Eizirik D.L. (2008). An update on lipotoxic endoplasmic reticulum stress in pancreatic beta-cells. Biochem. Soc. Trans.

[149] Cnop M., Ladrière L., Igoillo-Esteve M., Moura R.F., Cunha D.A. (2010). Causes and cures for endoplasmic reticulum stress in lipotoxic β-cell dysfunction. Diabetes Obes. Metab..

[150] Biden T.J., Boslem E., Chu K.Y., Sue N. (2014). Lipotoxic endoplasmic reticulum stress, β cell failure, and type 2 diabetes mellitus. Trends Endocrinol. Metab..

[151] Cunha D.A., Hekerman P., Ladrière L., Bazarra-Castro A., Ortis F., Wakeham M.C., Moore F., Rasschaert J., Cardozo A.K., Bellomo E. (2008). Initiation and execution of lipotoxic ER stress in pancreatic beta-cells. J. Cell. Sci..

[152] Cao S.S., Kaufman R.J. (2014). Endoplasmic reticulum stress and oxidative stress in cell fate decision and human disease. Antioxid. Redox Signal.

[153] Meyerovich K., Ortis F., Allagnat F., Cardozo A.K. (2016). Endoplasmic reticulum stress and the unfolded protein response in pancreatic islet inflammation. J. Mol. Endocrinol..

[154] Grootjans J., Kaser A., Kaufman R.J., Blumberg R.S. (2016). The unfolded protein response in immunity and inflammation. Nat. Rev. Immunol..

[155] Walter P., Ron D. (2011). The unfolded protein response: From stress pathway to homeostatic regulation. Science.

[156] Gardner B.M., Walter P. (2011). Unfolded proteins are Ire1-activating ligands that directly induce the unfolded protein response. Science.

[157] Novoa I., Zeng H., Harding H.P., Ron D. (2001). Feedback inhibition of the unfolded protein response by GADD34-mediated dephosphorylation of eIF2alpha. J. Cell Biol..

[158] Ma Y., Hendershot L.M. (2003). Delineation of a negative feedback regulatory loop that controls protein translation during endoplasmic reticulum stress. J. Biol. Chem..

[159] Urano F., Wang X., Bertolotti A., Zhang Y., Chung P., Harding H.P., Ron D. (2000). Coupling of stress in the ER to activation of JNK protein kinases by transmembrane protein kinase IRE1. Science.

[160] Maurel M., Chevet E., Tavernier J., Gerlo S. (2014). Getting RIDD of RNA: IRE1 in cell fate regulation. Trends Biochem. Sci..

[161] Yoshida H., Matsui T., Yamamoto A., Okada T., Mori K. (2001). XBP1 mRNA is induced by ATF6 and spliced by IRE1 in response to ER stress to produce a highly active transcription factor. Cell.

[162] Tsuru A., Imai Y., Saito M., Kohno K. (2016). Novel mechanism of enhancing IRE1α-XBP1 signalling via the PERK-ATF4 pathway. Sci. Rep..

[163] Gurzov E.N., Ortis F., Cunha D.A., Gosset G., Li M., Cardozo A.K., Eizirik D.L. (2009). Signaling by IL-1beta+IFN-gamma and ER stress converge on DP5/Hrk activation: A novel mechanism for pancreatic beta-cell apoptosis. Cell Death Differ..

[164] Gurzov E.N., Germano C.M., Cunha D.A., Ortis F., Vanderwinden J.M., Marchetti P., Zhang L., Eizirik D.L. (2010). p53 up-regulated modulator of apoptosis (PUMA) activation contributes to pancreatic beta-cell apoptosis induced by proinflammatory cytokines and endoplasmic reticulum stress. J. Biol. Chem..

[165] Ron D., Walter P. (2007). Signal integration in the endoplasmic reticulum unfolded protein response. Nat. Rev. Mol. Cell Biol..

[166] Cardozo A.K., Ortis F., Storling J., Feng Y.M., Rasschaert J., Tonnesen M., Van Eylen F., Mandrup-Poulsen T., Herchuelz A., Eizirik D.L. (2005). Cytokines downregulate the sarcoendoplasmic reticulum pump Ca^2+^ ATPase 2b and deplete endoplasmic reticulum Ca2+, leading to induction of endoplasmic reticulum stress in pancreatic beta-cells. Diabetes.

[167] Hara T., Mahadevan J., Kanekura K., Hara M., Lu S., Urano F. (2014). Calcium efflux from the endoplasmic reticulum leads to β-cell death. Endocrinology.

[168] Preston A.M., Gurisik E., Bartley C., Laybutt D.R., Biden T.J. (2009). Reduced endoplasmic reticulum (ER)-to-Golgi protein trafficking contributes to ER stress in lipotoxic mouse beta cells by promoting protein overload. Diabetologia.

[169] Li Y., Ge M., Ciani L., Kuriakose G., Westover E.J., Dura M., Covey D.F., Freed J.H., Maxfield F.R., Lytton J. (2004). Enrichment of endoplasmic reticulum with cholesterol inhibits sarcoplasmic-endoplasmic reticulum calcium ATPase-2b activity in parallel with increased order of membrane lipids: Implications for depletion of endoplasmic reticulum calcium stores and apoptosis in cholesterol-loaded macrophages. J. Biol. Chem..

[170] Marmugi A., Parnis J., Chen X., Carmichael L., Hardy J., Mannan N., Marchetti P., Piemonti L., Bosco D., Johnson P. (2016). Sorcin Links Pancreatic β-Cell Lipotoxicity to ER Ca^2+^ Stores. Diabetes.

[171] Lytrivi M., Ghaddar K., Lopes M., Rosengren V., Piron A., Yi X., Johansson H., Lehtiö J., Igoillo-Esteve M., Cunha D.A. (2020). Combined transcriptome and proteome profiling of the pancreatic β-cell response to palmitate unveils key pathways of β-cell lipotoxicity. BMC Genom..

[172] Hansen H.G., Schmidt J.D., Søltoft C.L., Ramming T., Geertz-Hansen H.M., Christensen B., Sørensen E.S., Juncker A.S., Appenzeller-Herzog C., Ellgaard L. (2012). Hyperactivity of the Ero1α oxidase elicits endoplasmic reticulum stress but no broad antioxidant response. J. Biol. Chem..

[173] Mehmeti I., Lortz S., Avezov E., Jörns A., Lenzen S. (2017). ER-resident antioxidative GPx7 and GPx8 enzyme isoforms protect insulin-secreting INS-1E β-cells against lipotoxicity by improving the ER antioxidative capacity. Free Radic. Biol. Med..

[174] Boslem E., MacIntosh G., Preston A.M., Bartley C., Busch A.K., Fuller M., Laybutt D.R., Meikle P.J., Biden T.J. (2011). A lipidomic screen of palmitate-treated MIN6 β-cells links sphingolipid metabolites with endoplasmic reticulum (ER) stress and impaired protein trafficking. Biochem. J..

[175] Boslem E., Weir J.M., MacIntosh G., Sue N., Cantley J., Meikle P.J., Biden T.J. (2013). Alteration of endoplasmic reticulum lipid rafts contributes to lipotoxicity in pancreatic β-cells. J. Biol. Chem..

[176] Acosta-Montaño P., Rodríguez-Velázquez E., Ibarra-López E., Frayde-Gómez H., Mas-Oliva J., Delgado-Coello B., Rivero I.A., Alatorre-Meda M., Aguilera J., Guevara-Olaya L. (2019). Fatty Acid and Lipopolysaccharide Effect on Beta Cells Proteostasis and its Impact on Insulin Secretion. Cells.

[177] Giacca A., Xiao C., Oprescu A.I., Carpentier A.C., Lewis G.F. (2011). Lipid-induced pancreatic β-cell dysfunction: Focus on in vivo studies. Am. J. Physiol. Endocrinol. Metab..

[178] Ly L.D., Ly D.D., Nguyen N.T., Kim J.H., Yoo H., Chung J., Lee M.S., Cha S.K., Park K.S. (2020). Mitochondrial Ca^2+^ Uptake Relieves Palmitate-Induced Cytosolic Ca^2+^ Overload in MIN6 Cells. Mol. Cells.

[179] Laurindo F.R., Araujo T.L., Abrahão T.B. (2014). Nox NADPH oxidases and the endoplasmic reticulum. Antioxid. Redox Signal.

[180] Gehrmann W., Würdemann W., Plötz T., Jörns A., Lenzen S., Elsner M. (2015). Antagonism Between Saturated and Unsaturated Fatty Acids in ROS Mediated Lipotoxicity in Rat Insulin-Producing Cells. Cell Physiol. Biochem..

[181] Malhotra J.D., Kaufman R.J. (2007). Endoplasmic reticulum stress and oxidative stress: A vicious cycle or a double-edged sword?. Antioxid. Redox Signal.

[182] Song B., Scheuner D., Ron D., Pennathur S., Kaufman R.J. (2008). Chop deletion reduces oxidative stress, improves beta cell function, and promotes cell survival in multiple mouse models of diabetes. J. Clin. Investig..

[183] Back S.H., Scheuner D., Han J., Song B., Ribick M., Wang J., Gildersleeve R.D., Pennathur S., Kaufman R.J. (2009). Translation attenuation through eIF2alpha phosphorylation prevents oxidative stress and maintains the differentiated state in beta cells. Cell Metab..

[184] Li J., Zhu H., Shen E., Wan L., Arnold J.M., Peng T. (2010). Deficiency of rac1 blocks NADPH oxidase activation, inhibits endoplasmic reticulum stress, and reduces myocardial remodeling in a mouse model of type 1 diabetes. Diabetes.

[185] Li G., Scull C., Ozcan L., Tabas I. (2010). NADPH oxidase links endoplasmic reticulum stress, oxidative stress, and PKR activation to induce apoptosis. J. Cell Biol..

[186] Kuwabara W.M., Zhang L., Schuiki I., Curi R., Volchuk A., Alba-Loureiro T.C. (2015). NADPH oxidase-dependent production of reactive oxygen species induces endoplasmatic reticulum stress in neutrophil-like HL60 cells. PLoS ONE.

[187] Görlach A., Bertram K., Hudecova S., Krizanova O. (2015). Calcium and ROS: A mutual interplay. Redox Biol..

[188] Contreras-Ferrat A., Llanos P., Vásquez C., Espinosa A., Osorio-Fuentealba C., Arias-Calderon M., Lavandero S., Klip A., Hidalgo C., Jaimovich E. (2014). Insulin elicits a ROS-activated and an IP_3_-dependent Ca^2+^ release, which both impinge on GLUT4 translocation. J. Cell Sci..

[189] Vilas-Boas E.A., Carlein C., Nalbach L., Almeida D.C., Ampofo E., Carpinelli A.R., Roma L.P., Ortis F. (2021). Early Cytokine-Induced Transient NOX2 Activity Is ER Stress-Dependent and Impacts β-Cell Function and Survival. Antioxidants.

